# Evaluation of Silicon Nitride as a Substrate for Culture of PC12 Cells: An Interfacial Model for Functional Studies in Neurons

**DOI:** 10.1371/journal.pone.0090189

**Published:** 2014-02-27

**Authors:** Johan Jaime Medina Benavente, Hideo Mogami, Takashi Sakurai, Kazuaki Sawada

**Affiliations:** 1 Department of Electrical and Electronic Information Engineering, Toyohashi University of Technology, Toyohashi, Japan; 2 Core Research for Evolutional Science and Technology Program, Japan Science and Technology Agency, Tokyo, Japan; 3 Faculty of Health Promotional Sciences, Hamamatsu University, Hamamatsu, Japan; 4 Electronics-Inspired Interdisciplinary Research Institute, Toyohashi University of Technology, Toyohashi, Japan; Emory University/Georgia Insititute of Technology, United States of America

## Abstract

Silicon nitride is a biocompatible material that is currently used as an interfacial surface between cells and large-scale integration devices incorporating ion-sensitive field-effect transistor technology. Here, we investigated whether a poly-L-lysine coated silicon nitride surface is suitable for the culture of PC12 cells, which are widely used as a model for neural differentiation, and we characterized their interaction based on cell behavior when seeded on the tested material. The coated surface was first examined in terms of wettability and topography using contact angle measurements and atomic force microscopy and then, conditioned silicon nitride surface was used as the substrate for the study of PC12 cell culture properties. We found that coating silicon nitride with poly-L-lysine increased surface hydrophilicity and that exposing this coated surface to an extracellular aqueous environment gradually decreased its roughness. When PC12 cells were cultured on a coated silicon nitride surface, adhesion and spreading were facilitated, and the cells showed enhanced morphological differentiation compared to those cultured on a plastic culture dish**.** A bromodeoxyuridine assay demonstrated that, on the coated silicon nitride surface, higher proportions of cells left the cell cycle, remained in a quiescent state and had longer survival times. Therefore, our study of the interaction of the silicon nitride surface with PC12 cells provides important information for the production of devices that need to have optimal cell culture-supporting properties in order to be used in the study of neuronal functions.

## Introduction

Current technological developments in bioengineering are providing new opportunities for cell biologists to develop new avenues of research and to investigate deeper into the molecular mechanisms of cell function. The semiconductor field is one of the areas within the discipline of electronic engineering whose interaction with cellular sciences has considerable potential to have great impact on society [1]. Moreover, the rapid progress in semiconductor research has stimulated interest in the biocompatibility of large scale integration (LSI) materials to improve instruments for the study of cells [2], [3], [4], [5]. LSI technology is now deeply involved in the development and production of highly sensitive biosensors, the demand for which has increased in recent years in many areas of cell biology [6] including those related to excitable cells such as neurons.

Silicon nitride (Si_3_N_4_) is a synthetic compound with a range of valuable mechanical, thermal and chemical properties [7], [8] that make it an ideal compound for the production of ion-sensing membranes for LSI devices using complementary metal oxide semiconductor (CMOS) [6] and charge-coupled device (CCD) technologies [9]. Si_3_N_4_ is the material of choice for the sensing area of ion-sensitive field-effect transistors (ISFET) [10] because of its many useful properties, particularly its long-term stability under atmospheric conditions and ability to act as a waterproofing agent. Various applications based on ISFET have been developed including pH sensors using standard CMOS processes [11], [12], [13] and CCD-based pH [9] and potassium ion [14] imaging sensors. Furthermore, research is currently underway into the use of new image sensor devices for detecting changes in the concentrations of ions whose movements across cell membranes play an essential role in many biological processes. A particular goal at present is to find a means of measuring the ionic status of the extracellular environment in real-time, which is a necessary step in understanding cellular activities. Such measurements will require the cells to be attached to a sensor surface; one of the materials being explored for this purpose is Si_3_N_4_.

The in vitro culture of primary cells or of a cell line requires conditioning the surface of the culture dish to facilitate growth of cells with a normal phenotype for as long as possible. Currently, cell culture procedures are being adapted for Si_3_N_4_ substrates to identify the conditions that provide the ideal interface for biotechnology-related experiments. Thus, although the characteristics of these substrates have not yet been fully determined, there is already evidence that they are potentially useful substrates for cell culture. Rak et al. [15] reported on the use of the semiconductivity of Si_3_N_4_ for the study of neuronal cells, such as cochlear nucleus cells. It was also demonstrated that 3T3L1 adipocytes and H9c2 cardiac myocytes can grow on an Si_3_N_4_ substrate [16]. Moreover, Hirata et al. reported that coating Si_3_N_4_ surface with poly-L-ornithine and laminin improves PC12 cell adhesion [17].

The PC12 cell line was derived from a rat adrenomedullary tumor and represents a neuronal model cell due to its property of acquiring the characteristics of sympathetic neurons when exposed to nerve growth factor (NGF) [18]. Under favorable conditions, it is possible to maintain PC12 cell cultures for months on poly-L-lysine (PLL)-coated plastic dishes [18], [19]; by contrast, neurons are sensitive to their environment and require strictly monitored conditions to grow normally as a primary culture. For this reason, PC12 cells became widely used as the subject for testing biomedical devices such as neurotransmitter-based neural prostheses [20] microfabricated using polymer-coated Si_3_N_4_ as the culture surface and interface material between cells and artificial chips designed for retinal prosthesis applications [21], [22] The PC12 cell/Si_3_N_4_ interaction was also examined by Nishiyama et al. when designing an inverted atmospheric scanning electron microscope for the observation of subcellular structures in open environment [23].

For biotechnologists, culturing PC12 cells on synthetic materials used for biosensing devices represents the first step on the way to the establishment of a neuronal biosensor system for studying the neuron at the single cell and molecular levels. Therefore, the aim of this research is to characterize the biophysical interactions between the cell and the surface material, and to examine the effects of a PLL-coated Si_3_N_4_ substrate on the culture of neuron-like cells (PC12 cells), which may be used as a stable cell model for the study of neuronal growth and differentiation, including axonal guidance [18].

In this paper, we report that PC12 cells can be successfully cultured on a PLL-coated Si_3_N_4_ surface prepared using the low-pressure chemical vapor deposition method. The surface properties of this material were characterized using contact angle measurements and atomic force microscopy. We also compared the growth of PC12 cells on a PLL-coated Si_3_N_4_ surface to a PLL-coated plastic tissue culture dish by examining the effect of the biointerface on cell adhesion and morphology. Finally, we stimulated cells with NGF, a protein that is expressed preferentially in neuronal tissues and is responsible for the survival, differentiation and functional activities of the neurons in the peripheral and central nervous system [24], in order to assess the differences between cells grown on an Si_3_N_4_ surface to those on a polystyrene culture dish. Quantification of the proportions of cells incorporating bromodeoxyuridine (BrdU) on an Si_3_N_4_ substrate under a variety of conditions provided insights into the causes of the different behavior of these cells on the tested surfaces.

## Materials and Methods

### Chemicals

Poly-L-lysine (PLL) and concanavalin A solutions were obtained from Sigma-Aldrich Co. (St. Louis, MO, USA). Polystyrene cell culture dishes were obtained from BD Biosciences (San Jose, CA, USA). pDsRed2-N1 plasmid was obtained from Clontech Laboratories, Inc. (Mountain View, CA, USA). Attractene (Qiagen, Venlo, The Netherlands) was used as the transfection reagent. Cells were grown in Dulbecco’s modified Eagle’s medium-high glucose (DMEM) obtained from Sigma-Aldrich Co. and supplemented with fetal bovine serum (FBS; Biological Industries, Kibbutz Beit Haemek, Israel) and horse serum (Sigma-Aldrich Co.). Phosphate buffered saline (PBS) and trypsin-EDTA solution were purchased from Invitrogen/Gibco (Carlsbad, CA, USA). Nerve growth factor of mouse origin (mNGF) was purchased from Alomone Labs Ltd. (Jerusalem, Israel). The BrdU flow kit for bromodeoxyuridine staining was used with the Alexa Fluor 488 mouse anti-BrdU antibody (both from BD Biosciences).

### Silicon Nitride Surface Experiments

#### Surface characterization

Topographical images of the Si_3_N_4_ surface coated with PLL were obtained using a BioScope II atomic force microscope (Veeco Instruments Ltd., Japan) on a NanoScope V system (Bruker Corporation, MA, USA) operated in tapping mode in aqueous solution. Si_3_N_4_ cantilevers with a nominal spring constant of 0.15 N/m were used. The 3D images were produced using NanoScope V 7.0 software. The scanning areas were 1 µm^2^ with a scan rate of approximately 1.0 Hz. These experiments were performed in collaboration with Toray Research Center, Inc. (Otsu, Japan).

Changes to the topography and morphology of the PLL coated surface due to hydrolytic degradation after 5 days in contact with the extracellular solution were characterized using data collected with an atomic force microscope and by comparing 3D images from samples after 1 and 5 days of incubation in the extracellular solution at 37°C in a 5% CO_2_ incubator.

The surfaces were further characterized by measuring the static contact angle between the water and the PLL-coated Si_3_N_4_ surface or the coated polystyrene culture dish; the measurements were made using the contact angle analyzer model Phoenix Alpha (S.E.O. Surface Electro Optics Co. Ltd., Suwon, Korea). Images were processed using the Surfaceware 7 ver.10.11 software provided by the instrument company. The drop used for the angle measurements contained 6.5 (±0.3) µl MiliQ water. Twenty individual samples tested in two trials were considered and the average and standard error of the measurements were calculated.

#### Preparation and coating of silicon nitride surface

N-type silicon wafers with <100> orientation and 3.65–4.58 Ωcm resistivity were obtained from Ryoko Sangyo Corporation (Tokyo, Japan). Before deposition, the 10.16 cm wafers were treated with HCl+H_2_O_2_ (3∶1) for 10 minutes and rinsed with distilled water for 10 minutes. The wafers were then treated with NH_4_OH+H_2_O_2_+distilled water (1∶1: 6) and HF+distilled water (1∶50), with washes in distilled water for 5∼10 minutes after each treatment. Si_3_N_4_ was deposited by low pressure chemical vapor deposition. The wafers were subjected to 808°C with SiH_2_Cl_2_ gas at a flow rate of 200 sccm and NH_3_ gas at a flow rate of 500 sccm, at a constant pressure of 33.33 Pa for 30 minutes. The final deposition had a thickness of 100 nm. It was cut before use for cell culture using laser dicing.

Coated Si_3_N_4_ surfaces were carefully prepared before being used as a substrate for cell culture. The Si_3_N_4_ surfaces were washed with water and detergent to clean them of organic residues, rinsed with abundant distilled water, and dried at room temperature. They were then UV irradiated overnight prior to coating with 0.01% or 0.05% (vol/vol) PLL. Coated surfaces were incubated at room temperature for 2 hours before rinsing three times in autoclaved distilled water. Finally, they were dried at room temperature.

Extracellular solution was prepared and added to dishes containing Si_3_N_4_ surfaces to immerse them in a typical cell culture environment. The surfaces were examined by atomic force microscopy at 1 and 5 days to determine the extent of hydrolytic degradation. The extracellular solution was composed of 140 mM NaCl, 5 mM KCl, 1 mM MgCl_2_, 2.5 mM CaCl_2_, 10 mM HEPES and 3 mM glucose, and adjusted to pH7.3 using an NaOH solution. All of these chemical reagents were obtained from Sigma-Aldrich Co.

### Cell Experiments

#### Cell line and culture

Rat pheochromocytoma cells (PC12), a stable cell line with neuronal differentiation properties, were kindly provided by Riken Cell Bank (Riken BioResource Center, Ibaraki, Japan) and cultured in DMEM supplemented with 10% FBS and 10% horse serum. PC12 cells were seeded onto an Si_3_N_4_ surface or a plastic dish, both of which had been coated with PLL. Cultures were maintained at 37°C with 5%CO_2_ in air, in a humidified incubator. Morphological differentiation of the cells was promoted by replacing the medium with fresh medium containing 50 ng/ml NGF in the presence or absence of FBS and horse serum. The medium was changed every 3 days and NGF was added if required.

#### Generation of the stably transfected reporter gene cell line

PC12 cells were used as host cells in transfection experiments. Transfection was carried out using an expression vector encoding the DsRed2 fluorescent protein in order to visualize cell morphology following seeding of the cells onto non-transparent substrates. Transfection was achieved using the Attractene transfection reagent according to the manufacturer’s protocol. DsRed2 transfected cells were selected using the functional expression of the neomycin resistance gene included in the plasmid sequence. Cells were viewed using an Olympus BX51 fluorescence microscope (Olympus Corporation, Tokyo, Japan) and images were captured with a Neo sCMOS camera (Andor Technology plc, Belfast, Northern Ireland). DsRed fluorescent protein and its variant DsRed2 have been widely used as markers of gene expression and protein localization due to their usefulness for in vivo labeling of mammalian cells [25] including PC12 cells [26], [27], [28], [29].

#### Cell counts

PC12 cells (7×10^4^ cells/ml) were seeded onto a sterilized PLL coated Si_3_N_4_ surface located on a 35 mm plastic dish or onto a polystyrene culture dish. Cells were incubated under the conditions described above for various lengths of time. Cell counts were made on cell suspensions obtained by carefully aspirating the culture medium and then harvesting cells after trypsinization (0.025% trypsin-EDTA) using a slow manual pipetting technique. The collected cells were transferred to a new tube, the suspension thoroughly mixed, and an aliquot of predetermined volume was placed into an improved Neubauer counting chamber in order to quantify the number of cells. The polystyrene dishes used for the comparison were composed of standard plasticware material used to culture cells in cell biology research.

### Measurement of Cell Proliferation

PC12 cell proliferation was assessed using the bromodeoxyuridine (BrdU) labeling index. Cells attached to Si_3_N_4_ or polystyrene surfaces were incubated in growth medium containing a 10 µM BrdU solution for 2 hours at 37°C. The medium was removed and the cells were fixed by adding a fixation buffer containing 4% paraformaldehyde (wt/vol) for 20 minutes at room temperature. The cells were washed in PBS and permeabilized with Perm Buffer III for 10 minutes at room temperature. They were then washed in PBS and blocked by adding an FBS-based staining buffer for 1 hour. After treatment with a DNase I solution for 1 hour at 37°C, the cells were washed in PBS and incubated with Alexa Fluor 488 anti-BrdU antibody (1∶40) diluted in the staining buffer for 90 minutes. The cells were then washed and counterstained with Hoechst 33342. Si_3_N_4_ surfaces were attached to a glass slide and mounted with a coverslip. To prevent drying due to the thickness of the Si_3_N_4_ surface, the cells were visualized and analyzed immediately after sample preparation. Cells cultured in medium lacking BrdU were used as negative controls.

### Statistical Analysis

The XLSTAT software version 2013.6.03 for Windows (Addinsoft, New York, USA) was used for analysis. Using Shapiro-Wilk and Anderson-Darling tests, we determined that the data used came from a normally distributed population. Statistically significant differences between sample groups were identified using an independent *t-*test for all samples against the control. According to the *p* value found, the null hypothesis was accepted or rejected; in the latter case we concluded that the difference between the groups tested under different experimental conditions was statistically significant; *p*⩽0.05 was regarded as statistically significant, *p*⩽0.01 as highly significant and *p*⩽0.001 as very highly significant. Additionally, when a significant difference was found, the statistical power of the *t-*test for two independent groups was analyzed using Power and Precision software version 4.1.0 (Biostat, Englewood, NJ, USA) concluding that in this situation, 100% of studies would be expected to yield a significant effect, rejecting the null hypothesis that the population means are equal. The Mann Whitney U test was applied when the sample size was equal or less than 10.

## Results

### Attachment of PC12 Cells to the Silicon Nitride Surface

**Figure 1:**
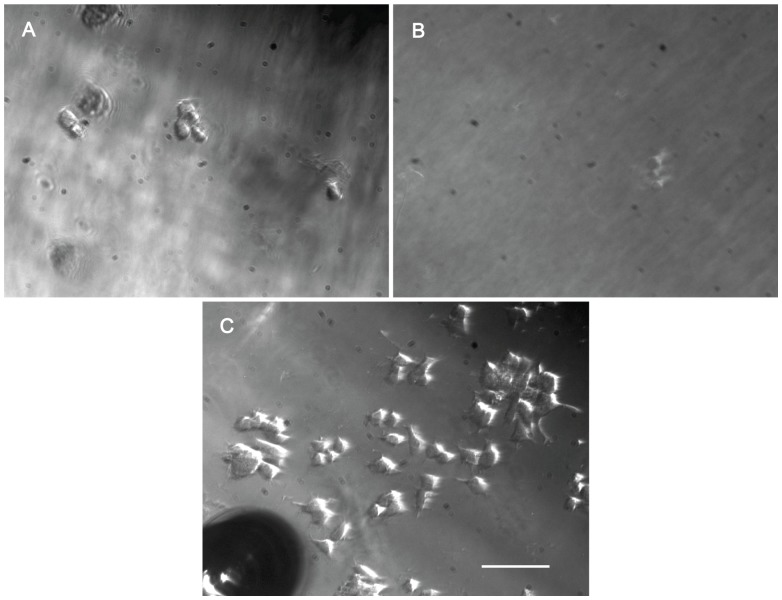
PC12 cell adhesion to an Si_3_N_4_ surface. PC12 cells were seeded at the same concentration on Si_3_N_4_ surfaces coated with different cell adhesion molecules and images of representative areas were captured using an inverted microscope 24 hours later. Non-coated Si_3_N_4_ surfaces were used as a control. (A) Non-coated Si_3_N_4_ surface, (B) concanavalin A and (C) 0.01% poly-L-lysine coated Si_3_N_4_ surface. (Scale bar: 50 µm).

The substrate under test, Si_3_N_4_, was conditioned by coating with cell adhesive polymers to facilitate cell attachment and growth. Two polymers were tested, concanavalin A (10 mg/ml) and PLL (0.01%), and analyzed using PC12 cells. A non-coated Si_3_N_4_ surface was used as the control. We assessed the morphological appearance of the cells to define the differences in attachment between the surface samples. Immediately after seeding, the cells had a small, rounded shape on all the tested surfaces. At 24 hours after seeding on the PLL-coated surface, they had a flattened appearance with small cytoplasmic projections; this alteration indicated cell adhesion to this surface [30], [31], [32]. By contrast, cells on the concanavalin A coated surface showed little to no adhesion and resembled the control (Fig. 1); the cells that did adhere showed little evidence of cytoplasmic spreading. Thus, attachment of PC12 cells to the Si_3_N_4_ surface was improved by coating with 0.01% PLL, which stimulated a higher rate of cell adhesion and showed little indication of the toxicity of the concanavalin A solution.

### Characterization of the Silicon Nitride Surface

**Figure 2:**
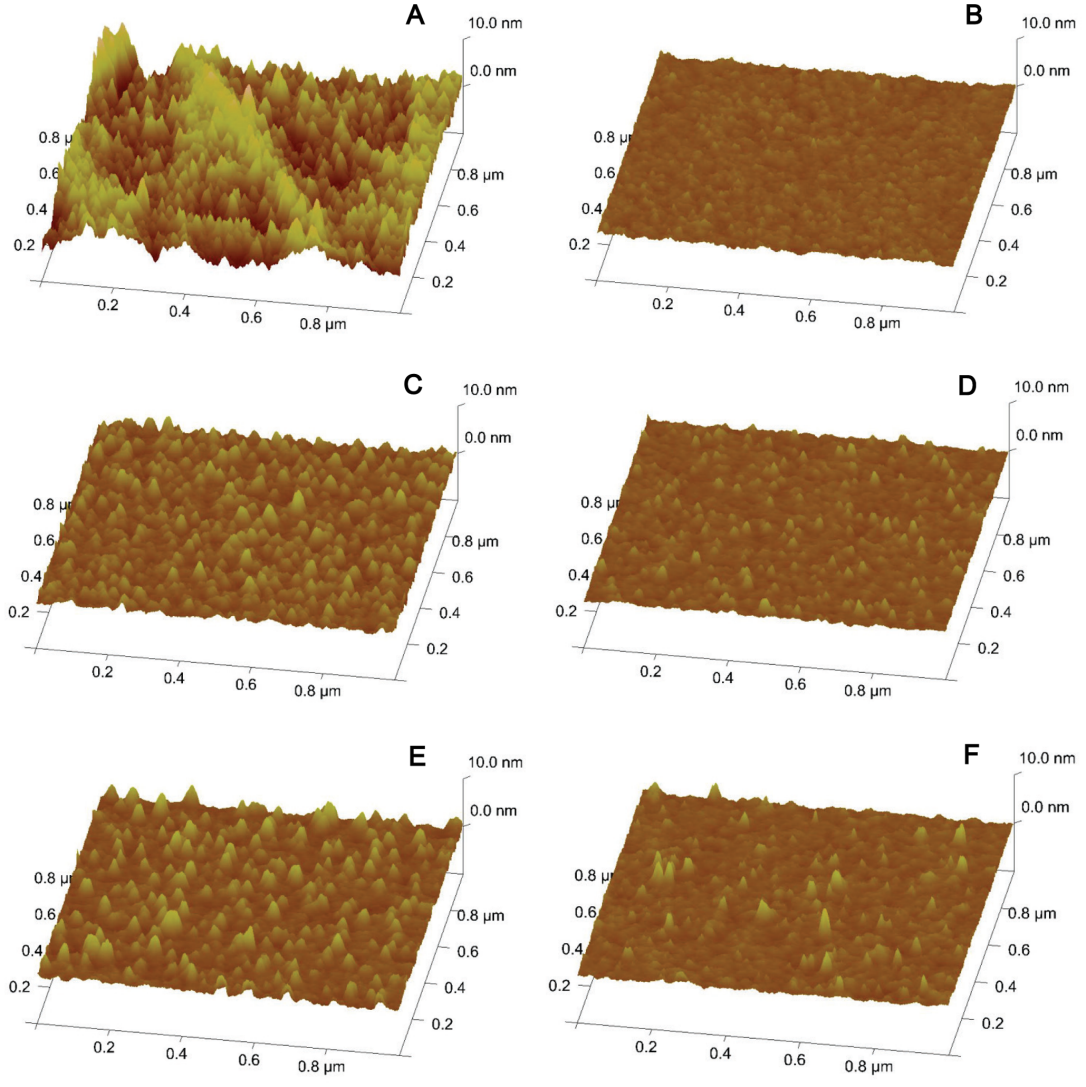
Surface structure analysis of an Si_3_N_4_ surface under different coating conditions. Three-dimensional atomic force microscope height images. (A) 0.01% poly-L-lysine coated plastic dish, (B) non-coated Si_3_N_4_ surface, (C) 0.01% poly-L-lysine coated Si_3_N_4_ surface at 1 day after coating and (D) at 5 days after coating, (E) 0.05% poly-L-lysine coated Si_3_N_4_ surface at 1 day after coating and (F) at 5 days after coating.

The surface topographies of Si_3_N_4_ samples were analyzed using atomic force microscopy. The non-coated Si_3_N_4_ surface was found to be relatively smooth across the 1 µm^2^ scan area and its root mean squared roughness (Rq) was 0.342±0.1 nm under aqueous conditions. The roughness of the Si_3_N_4_ surface was measured after coating with two different concentrations of PLL (0.01% and 0.05%). Due to this procedure, the Rq of the examined surfaces increased to 0.626±0.1 nm for 0.01% PLL and 0.791±0.1 nm for 0.05% PLL. Furthermore, the elaboration of data deriving from atomic force microscopy examination of the surfaces indicated a difference between the Rq of the 0.01% PLL coated Si_3_N_4_ surface and the 0.01% PLL coated plastic dish (Rq = 2.36±0.5) which had a statistical significance determined using the Mann-Whitney U test (*p<*0.05); the coated surface topography of the latter reached very high parameter values. PLL aggregates were not observed on the Si_3_N_4_ surface even when high concentrations of the polymer were used. After 5 days of immersion at 37°C in the extracellular solution, Si_3_N_4_ surfaces coated with PLL underwent hydrolytic degradation in which the coated surface featured peaks flattened to an Rq of 0.374±0.07 nm for 0.01% PLL and 0.530±0.12 nm for 0.05% PLL (Fig. 2).

**Figure 3:**
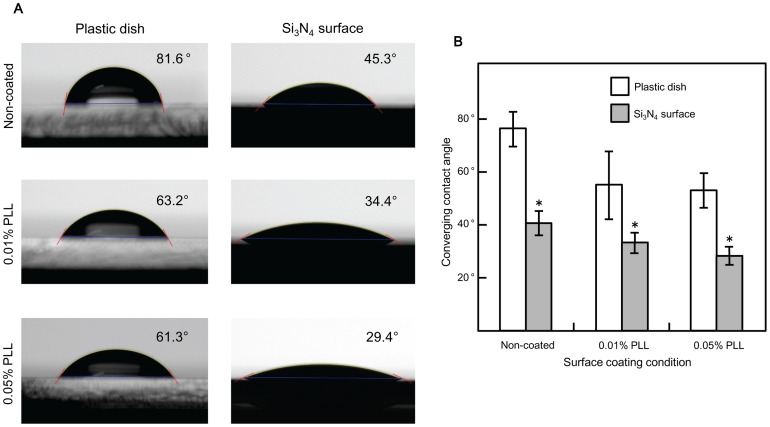
Analysis of the effect of poly-L-lysine coating on contact angles. (A) Substrates were coated with poly-L-lysine solution for 2 hours and then rinsed with distilled water. After overnight drying (∼16 hours) at room temperature, images were captured using a contact angle analyzer. The angle was calculated using the instrument software and the values shown represent the averages of the angles from both sides of the droplet. Non-coated surfaces were also tested for comparative purposes. Each picture is representative for each sample group. The light square observed in the center of the droplet of the pictures taken to the plastic dish samples corresponds to the area where light passes perpendicularly through the water so that refraction does not happen, a similar effect than the one produced by a spherical lens when illuminated. It is important to mention that the light source was located at the opposite side of the water droplet in relation to the camera lens. (B) Plastic culture dishes and Si_3_N_4_ surfaces were left untreated or coated with 0.01% or 0.05% poly-L-lysine. A contact angle analyzer was used to measure sessile drop contact angles. Values are the mean ± S.E. for twenty samples (Two trials of ten samples each). *, *p<*0.001 *vs.* plastic culture dish.

The wettability of the surfaces to water was determined using static contact angle measurements. We found that water contact angles differed between Si_3_N_4_ surfaces coated with different concentrations of PLL and also between Si_3_N_4_ surfaces and plastic dishes coated with the same PLL concentration (Fig. 3A). The untreated polystyrene culture dish was more hydrophobic than the untreated Si_3_N_4_ surface: plastic dish, 75.6° ±6.9; Si_3_N_4_ surface, 40°±4.1. PLL treatment reduced the contact angle in a concentration-dependent manner on Si_3_N_4_ surfaces: 0.01% PLL showed a contact angle of 33.9° ±3; 0.05% PLL had a contact angle of 27.8° ±3.2. However, no significant change was observed on the plastic dish: 0.01% PLL had a contact angle of 55.2° ±12.4; 0.05% had a contact angle of 52.8° ±6.8 (Fig. 3B). Thus, PLL increased the hydrophilicity of both surfaces but increasing the PLL concentration had no significant effect on the liquid-solid surface interaction on plastic dishes.

### PC12 Cell Attachment and Growth on a Poly-L-lysine Coated Silicon Nitride Surface

As PC12 cells were previously transfected with a plasmid encoding DsRed2 fluorescent protein for visualization matters, an additional experiment was performed in order to verify that the expression of the transfected reporter gene does not affect the morphology and behavior of PC12 cells. The obtained results are shown in Fig. S1.

Cell attachment and growth on the PLL coated Si_3_N_4_ surface were assessed by analysis of cell shape and the increase in the number of cells. The cells were cultured in the presence of serum and absence of NGF, which enabled their continuous proliferative activity. Cell growth rates were estimated using PC12 cells cultured on PLL coated Si_3_N_4_ surfaces or plastic cell culture dishes and counting cell numbers every other day for 9 days.

**Figure 4:**
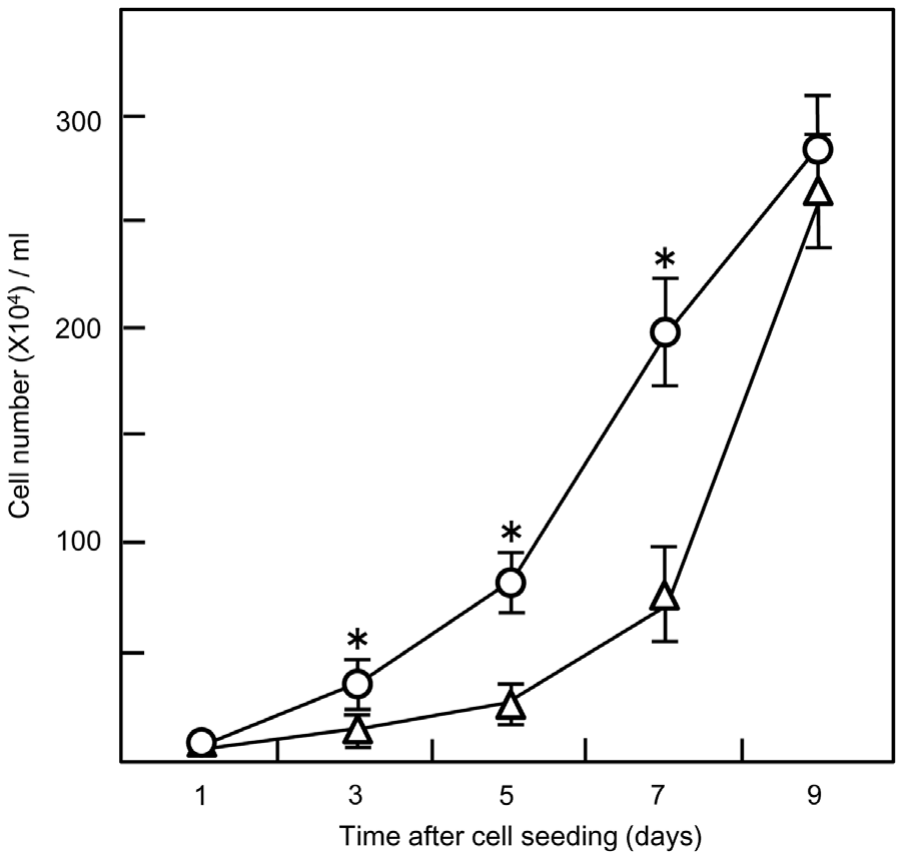
Non-NGF stimulated PC12 cell proliferation on different substrates. PC12 cells were seeded on 35 mm^2^ poly-L-lysine coated Si_3_N_4_ surfaces (○) or plastic culture dishes (▵) and counted every 2 days. The cells were cultured in DMEM +10% FBS +10% horse serum. The medium was replaced every 3 days. Values are the mean ± S.E. for twenty samples (Two trials of ten samples each). *, *p<*0.001 *vs.* plastic culture dish.

**Figure 5:**
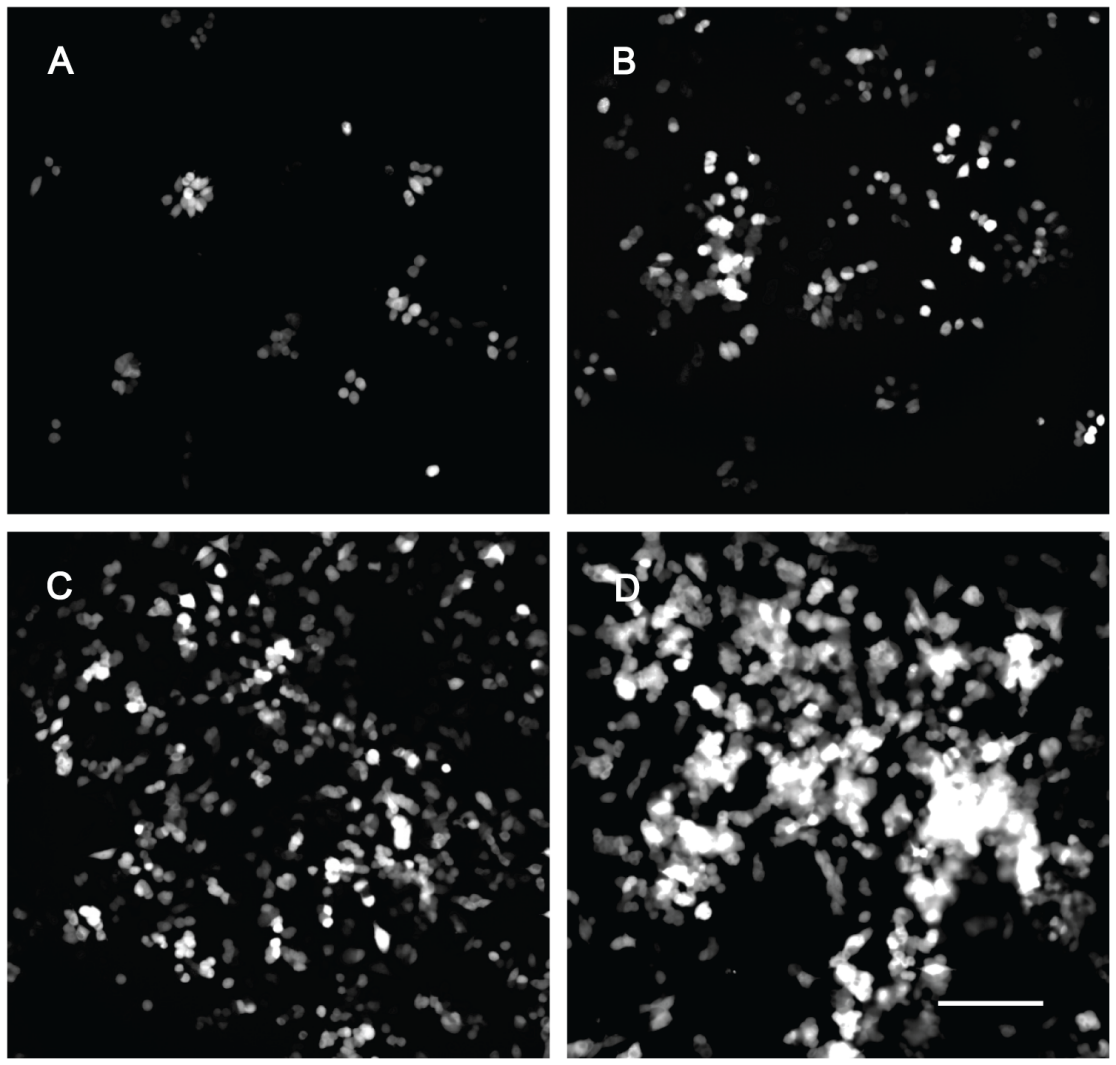
PC12 cell proliferation on a poly-L-lysine coated Si_3_N_4_ surface. PC12 cells expressing DsRed2 protein were seeded at a concentration of 7×10^4^ cells/ml on poly-L-lysine coated Si_3_N_4_ surfaces and images were captured every 2 days using a fluorescence microscope. (A) 3 days, (B) 5 days, (C) 7 days and (D) 9 days after cell seeding. Scale bar: 100 µm.

We found that the proliferation rate of cells cultured on the PLL coated Si_3_N_4_ surface was higher than in the plastic dish during the initial phase of the experiment (Fig. 4). On day 3, there were statistically more cells on the Si_3_N_4_ surface (34.99±8.3×10^4^ cells/ml ) than in the plastic dish (13.48±5.1×10^4^ cells/ml) and this difference was maintained until day 7 (Si_3_N_4_ surface, 197.94±24.1×10^4^; plastic dish, 73.85±9.6×10^4^ cells/ml). However, cells cultured on the coated plastic dish showed rapid proliferation between days 7 and 9, whereas, the proliferation rate decreased on the Si_3_N_4_ surface. As a result, there was no significant difference in cell numbers at day 9 between the substrates (Si_3_N_4_ surface, 280.02±27.7×10^4^; plastic dish, 261.93±20×10^4^ cells/ml). Neither tested surface showed evidence of cytotoxic effects and the flattened morphology of the PC12 cells during the 9 days of the experiment confirmed their improved attachment to PLL coated Si_3_N_4_ surface. This improvement can be seen in the DsRed2-transfected PC12 cells shown in Fig. 5, which displayed the characteristic polygonal shape and monolayer growth.

### Effect of FBS and NGF on the Proliferation of PC12 Cells on a Poly-L-lysine Coated Silicon Nitride Surface

Fetal bovine serum (FBS) is commonly used for cell culture. It contains growth factors and a rich variety of proteins that help the cells survive, grow and divide. Here, we examined the use of FBS as a variable affecting cell proliferation. We also used NGF to promote the differentiation of PC12 cells into neuron-like cells when cultured on a PLL coated Si_3_N_4_ surface. A preliminary experiment using 25, 50, and 100 ng/ml NGF showed that 50 ng/ml was sufficient to induce PC12 cells to differentiate into neuron-like cells (data not shown).

**Figure 6:**
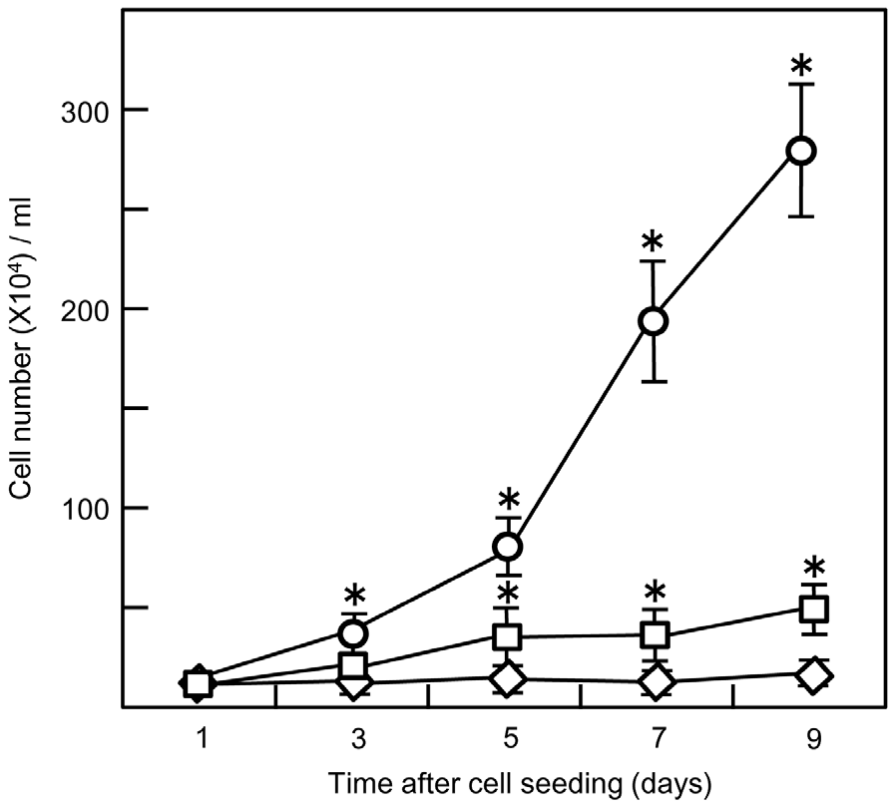
Effect of FBS and NGF on PC12 cell proliferation on a poly-L-lysine coated Si_3_N_4_ surface. PC12 cells were seeded onto poly-L-lysine coated Si_3_N_4_ surfaces in serum-supplemented growth medium and after 24 hours, cells were stimulated with NGF (50 ng/ml) in the presence/absence of FBS+horse serum. Cells were counted every 2 days. Three treatment groups were established: FBS (+) NGF (−) (○); FBS (+) NGF (+) (□); and FBS (−) NGF (+) (⋄). The values shown are the mean ± S.E. for twenty samples (Two trials of ten samples each). The presence of FBS significantly increased the cell proliferation rate (*, *p<*0.001 *vs.* FBS (−) NGF (+)); NGF significantly decreased the cell proliferation rate (*, *p<*0.001 *vs.* FBS (+) NGF (+)).

The supplementation of medium with both FBS and NGF reduced the rate of proliferation of PC12 cells on an Si_3_N_4_ surface compared to cultures with only FBS. The difference between the two groups of cultures reached significance at 3 days after plating the cells (NGF-absence, 34.99±8.3×10^4^ cells/ml; NGF-presence, 18.83±4.6×10^4^ cells/ml) and continued to be highly significant on subsequent days of culture. The increase in cell number in FBS(+)NGF(+) cultures was just 18% of that in FBS(+)NGF(−) cultures on the final day of the experiment (NGF-absence, 280.02±27.7×10^4^ cells/ml; NGF-presence, 51.09±9.3×10^4^ cells/ml). The increase in cell numbers in FBS(+)NGF(+) cultures occurred gradually although between 5 and 7 days after cell seeding, a significant variation was not observed. Our results show that PC12 cells cultured in FBS(−)NGF(+) did not proliferate but started to differentiate into neuron-like cells with pronounced neurite formation even after 1 day of NGF stimulation. The differences in cell numbers were very highly significant even after 3 days of culture (FBS-presence, 18.83±4.6×10^4^ cells/ml; FBS-absence, 8.58±2.2×10^4^ cells/ml) and this statistical significance was maintained until the last day of the experiment compared to cells in FBS(+)NGF(+) cultures (FBS-presence, 51.09±9.3×10^4^ cells/ml; FBS-absence, 15±3.9×10^4^ cells/ml). These data are graphically illustrated in Fig. 6.

**Figure 7:**
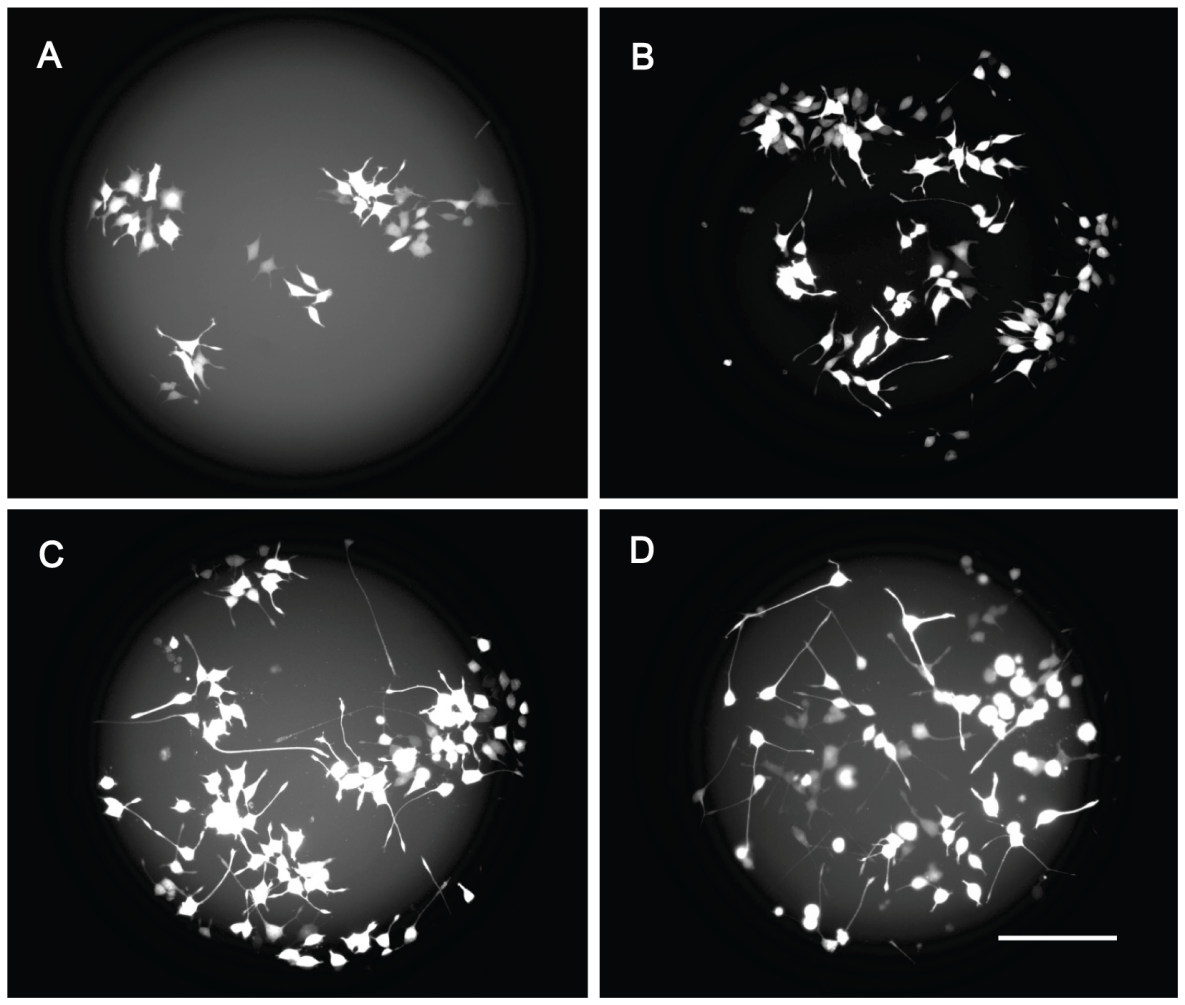
PC12 cell differentiation on a poly-L-lysine coated Si_3_N_4_ surface after NGF stimulation. PC12 cells expressing DsRed2 protein were seeded onto poly-L-lysine coated Si_3_N_4_ surfaces and 24 hours later (considered time for cell attachment), NGF (50 ng/ml) was added to the medium in the absence of FBS and horse serum. Images were captured using a fluorescence microscope at (A) 1 day, (B) 3 days, (C) 5 days and (D) 7 days after NGF stimulation. Scale bar: 100 µm.

The induction of neuritic extensions from differentiating PC12 cells by NGF was assessed by analyzing the morphology of cells grown under FBS(−)NGF(+) culture condition (Fig. 7). To aid visualization of PC12 cells on the Si_3_N_4_ surface, the cells were transfected with a plasmid carrying a red fluorescent protein coding sequence (pDsRed2-N1). At 1 day after NGF stimulation, the PC12 cells had attached to the PLL coated Si_3_N_4_ surface and minor processes, typically two to four in number (15–25 µm), had emerged from the soma of many cells. Although most of the cells presented outgrowth extensions, in general the cells still retained their neuronal-like symmetry.

At 3 days after NGF stimulation, the minor processes could be seen to have continued elongation; in some cells, a single extension was clearly longer than the others. By this time, the processes had reached lengths of up to 65 µm although symmetry between the soma extensions was still present in most of cells. At 4 and 7 days, the processes emerging from the soma were seen to have elongated with culture time (Fig. 7). In some cells, a single outgrowth extension had become predominant giving the cell an appearance similar to a developed neuron although lacking the neuronal branching. By 7 days, every cell had commenced differentiation although there were inter-cell differences in the timing of the morphological changes. Thus, the cultures contained cells with morphologies similar to those observed at earlier culture times alongside cells with long neurite-like processes. At the end of the culture period, extensions in the range 140 to 250 µm were observed. Cells that were isolated from contact with others had longer extensions than cells in accumulations.

### Growth of Extensions from PC12 Cells on Poly-L-lysine Coated Surfaces after NGF Stimulation

**Figure 8:**
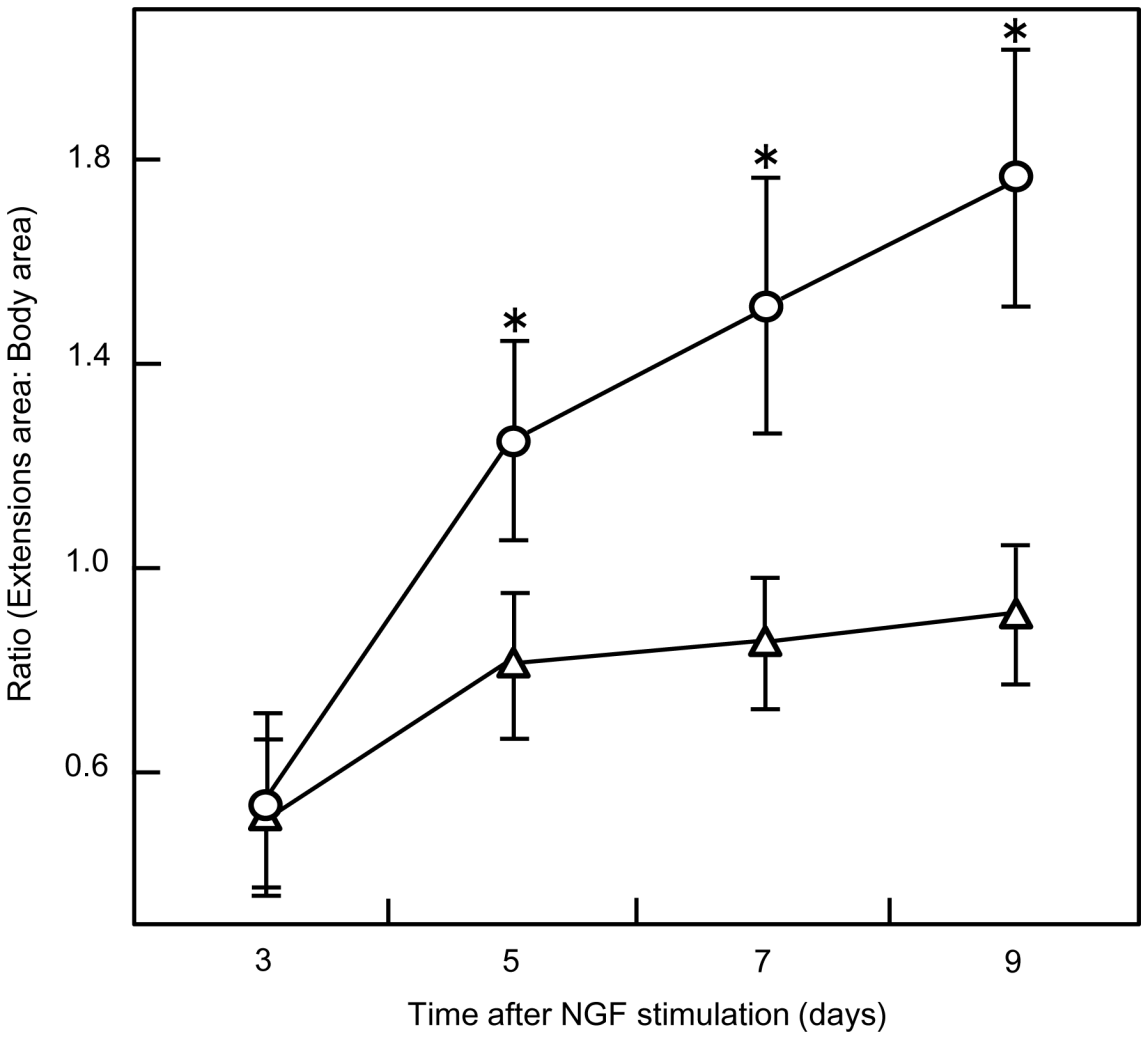
Growth of extensions in PC12 cells after NGF stimulation. PC12 cells seeded on different substrates were stimulated with NGF (50 ng/ml) in the absence of FBS and horse serum and images were captured every 2 days beginning at 3 days after NGF stimulation. Using AquaCosmos analysis software, the areas of differentiated cell bodies were compared to the areas of the outgrowth extensions. Representative data are shown in the graph. Cells cultured on an Si_3_N_4_ surface (○ ) or on a plastic culture dish (▵). The values shown are the mean ± S.E. of 30 samples. (*, *p<*0.001 *vs.* plastic culture dish).

Next we investigated the effect of the cell-substratum interface on neurite development in cells maintained under FBS-absence culture condition plus NGF. To quantitatively assess the morphological differentiation of PC12 cells, we calculated the ratio of the area of the neurite-like extensions (abbreviated here to neurites) to that of the cell body using AquaCosmos Software (Hamamatsu Photonics, Hamamatsu, Japan). In order to perform a standardized delimitation of the areas in all samples, we defined the limits of the cell soma based on the description of the contour determination of the basal soma area described by Happel et al. [33] applied to cells with multiple extensions and then we performed a boundary delineation of the extensions in all selected single cells. Thirty cells were randomly chosen at each time point and the data were plotted (Fig. 8). In a preliminary experiment to establish a negative control, we found that small filopodial protrusions were generated when PC12 cells were cultured for a long time without NGF stimulation (data not shown). For that reason we developed a proportional index to avoid the inclusion of cells with minor morphological changes stimulated by the intracellular production of growth factors. This index was defined as follows: if the summed area of all the neurites divided by that of the soma area gave a ratio >0.6, then we concluded that the cells had undergone morphological differentiation; if the value was <0.6, then it was possible that elongation of the extensions might not be a result of the application of NGF (Fig. S2).

Our analysis showed a faster and higher increase in the ratios of extension area: body area in cells cultured on a PLL coated Si_3_N_4_ surface. When cells were plated on the PLL coated Si_3_N_4_ surface, the selected neurons possessing representative lengths of neurites had an extension area that exceeded the size of the soma area after 5 days of culture. This relationship changed further by the next day and reached values up to 222% higher than the calculated ratios in the first days of the experiment. With time in culture, the ratios (a variable representing the length and number of neurites) increased and showed a significant difference to the samples grown on plastic dishes (Fig. 8). The increase in the calculated ratios in PC12 cells cultured on plastic dishes was not significant and amounted to only a 66% increase between the first and last days of the experiment; moreover, cell body areas were larger than the sum of all the areas of neurites in all of samples seeded on plastic dishes up to the end of the experiment when most of the outgrowth extensions were at their maximal lengths. The cells growing in the plastic dishes also developed extensions that grew during the first five days after NGF stimulation but then remained almost constant in size until the end of the experiment.

### PC12 Cell Proliferation on a Poly-L-lysine Coated Silicon Nitride Surface

**Figure 9:**
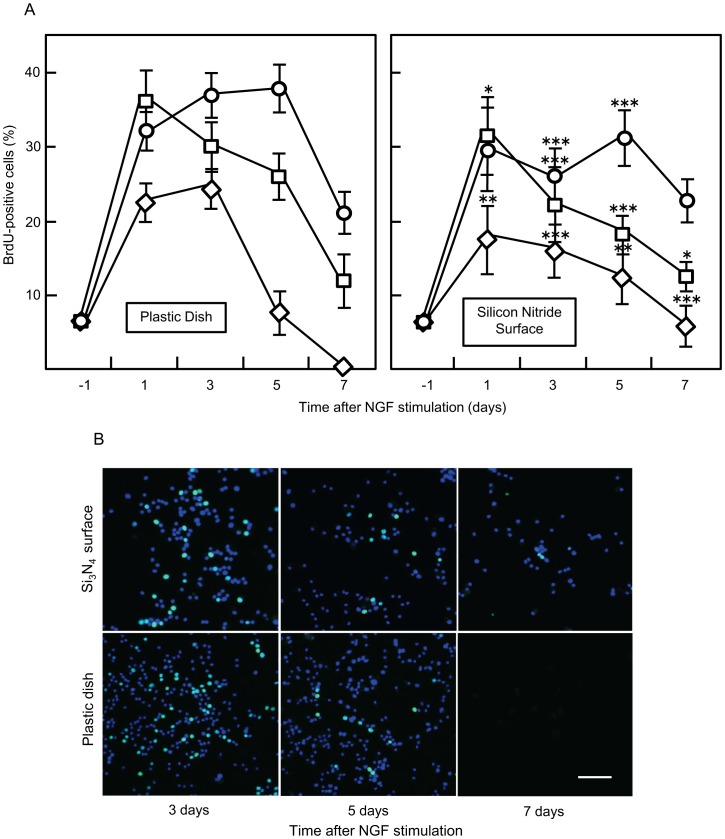
Assessment of PC12 cell proliferation using a BrdU assay. (A) Cells were seeded on different surfaces and 24 hours after seeding, they were stimulated with NGF (50 ng/ml). After 2 hours incubation in growth medium containing BrdU solution, cells were fixed, permeabilized and stained using an Alexa Fluor 488 conjugated anti-BrdU antibody. Finally, BrdU positive cells were counted in 20 samples and the proportion (%) of labeled cells per counted field was calculated. Standard error is shown using error bars. The following media were tested: FBS (+) NGF (−) (○); FBS (+) NGF (+) (□); and FBS (−) NGF (+) (⋄). (*, *p<*0.05; * *, *p<*0.01; * * *, *p<*0.001 *vs.* plastic culture dish). (B) Cells were seeded on different surfaces and cultured in the absence of serum and with NGF treatment. They were fixed using a paraformaldehyde-based solution and immunocytochemical detection of the BrdU antigen was performed to identify proliferative (S phase) cells. Images were captured at different time points. Representative images of each condition are shown. Scale bar: 50 µm.

We assessed cell proliferation using a BrdU assay, which identifies dividing cells following incorporation of BrdU during the DNA synthesis (S phase) of the cell cycle. PC12 cells were seeded under identical conditions on both tested surfaces 1 day before NGF stimulation; this timing for PC12 cell seeding/stimulating was based on preliminary experiments using an Si_3_N_4_ surface. The stimulative effect of FBS on proliferation was partially inhibited by the contact of PC12 cells with the Si_3_N_4_ surface (Fig. 9A). In the presence of FBS (and disregarding the addition of NGF), the number of BrdU-positive cells after 8 days of culture did not differ significantly between the two substrates; however, cells cultured on the plastic dish in the presence of NGF reached their peak rate of proliferation earlier than cells cultured on the same surface in the absence of NGF. PC12 cells cultured on the Si_3_N_4_ surface showed their peak rate of proliferation during the first days when cultured in the presence of NGF; thereafter, the rates of proliferation were lower than in cultures without NGF (Fig, 9A). In cultures without FBS but with NGF, the PC12 cells showed a greater rate of survival and morphological differentiation on the Si_3_N_4_ surface compared to plastic dishes. Although the peak level of PC12 cell proliferation was lower on the Si_3_N_4_ surface in FBS(−)NGF(+) cultures, the rate of decrease of BrdU-positive cells was lower than in cultures on plastic dishes. Survival of the seeded PC12 cells was significantly reduced by interaction with the plastic dish substrate, the absence of FBS, and the presence of NGF (especially after 3 days of NGF stimulation). By contrast, cells seeded on the Si_3_N_4_ surface proliferated at a slower rate but survived longer; although their proliferation rate decreased considerably at the longer culture times, viable cells were still detected after 7 days of NGF stimulation (Fig. 9B).

## Discussion

As Si_3_N_4_ is a biocompatible non-oxide ceramic and is also the main waterproofing compound used in the conventional LSI process, it has become the material of choice for the sensing surface of cell culture-supporting bioimaging sensor devices. Untreated Si_3_N_4_ is unable to promote cell attachment or proliferation, which represents a major limitation for its use in the fabrication of bioengineering instruments. This limitation can be overcome through conditioning the Si_3_N_4_ by use of a functional surface coating. In the present study, we demonstrate that adhesion and proliferation of PC12 cells were significantly improved by the PLL coating of the Si_3_N_4_ surface. Even concanavalin A, a lectin used to strengthen cell adhesion to the substratum [34], showed a lower adhesive effect than PLL when used on an Si_3_N_4_ surface. This fact may be explained by the difference in the nonspecific adsorption of the coating to the Si_3_N_4_ surface. The promotion of cell adhesion on the Si_3_N_4_ surface by the PLL coating could be the result of the enhancement of electrostatic interactions between the negatively charged ions of the cell membrane and the positively charged amino groups in the side chain of the lysine polymer. The same mediating effect of PLL may facilitate the attachment of PC12 cells to polystyrene dish which in non-coated conditions, showed to be an unsuitable substrate for the culture of this cell line (Fig. S4) as it was also reported by Fujii et al. [30].

Another factor that influences PLL enhancement of cell adhesion is the hydrophilicity of the surface although it is important to remark that cell interaction with PLL layer may as well be affected by the topographical features of the coated surfaces which are not identical. Root mean square roughness value of 0.01% PLL coated plastic dish was found to be almost four times higher than when measured on the Si_3_N_4_ surface coated with the same PLL concentration which may facilitate cell attachment on the plastic dish. However, the AFM height images revealed a higher conformational flexibility of the PLL coated plastic surface which was not uniformly distributed in comparison with the continuously covered and flatter layer produced by the PLL coating of the Si_3_N_4_ surface (Fig. 2). From our contact angle analysis, we found that cell adhesion was enhanced and that the average contact angle value decreased from 40.04° to 33.89° when the Si_3_N_4_ surface was coated with 0.01% PLL. Our results also showed that non-coated plastic culture dishes were considerably less hydrophilic than the non-coated Si_3_N_4_ surface, even though plasma treatment was used for their preparation in order to generate randomized chemical groups that remained covalently attached to the polystyrene surface turning it into a charged hydrophilic material [35]. The addition of 0.01% PLL increased the hydrophilicity of both substrates, with the Si_3_N_4_ surface having the higher tendency to adsorb water than the polystyrene surface of the plastic dish in both tested coating conditions. This fact implies the adhesion of water molecules to PLL coated Si_3_N_4_ surface without the permeation of the substance into this material which is considered a passivation layer for biotechnological applications [36]. Increasing the PLL concentration to 0.05% had no significant effect on the wettability of the plastic dish but did make the Si_3_N_4_ surface more hydrophilic and decreased the average contact angle to less than 30°. As shown in Fig. 2, the PLL coated Si_3_N_4_ surface was not stable throughout the experiment. Our atomic force microscopy analysis indicated that hydrolytic degradation occurred during a 5 day culture period due to continuous contact with the extracellular solution. We found that the mean roughness of a 0.01% PLL coating fell to approximately 44% after 5 days; this decrease affected cell adhesive conditions as it was confirmed in the results shown in Fig. S3, where it can be observed that more cells were attached to one day degraded PLL coated than to five days degraded PLL coated Si_3_N_4_ surfaces after one day of culture which may have also influenced cell behavior.

Glass dishes were also tested as a cell culture substrate in order to establish a comparison between two surfaces with similar hydrophilic properties. Our results showed that the wettability of uncoated Si_3_N_4_ and glass surfaces, determined by measuring the contact angle, was not significantly different (data not shown); however glass surface supported PC12 cell culture in the absence of PLL coating (Fig. S4). This fact may be mediated not just by the different roughness of both materials that may affect the cells directly and immediately after seeding but by the contact of the extracellular environment with these surfaces whose properties may have a different enhancive effect on the adsorption of active proteins from the serum in the culture medium [37], having a different impact on the initial attachment of the cells.

Our results also showed that the interaction between the Si_3_N_4_ surface and PC12 cells was also involved in cell spreading in medium supplemented with serum. As the non-coated Si_3_N_4_ surface was more hydrophilic than the non-coated plastic dish, attachment of the cells to the former might be facilitated by the interaction of the cells with the substrate. This fact may promote an earlier onset of proliferation on the Si_3_N_4_ surface. In the same way, the hydrolytic degradation of the PLL coating on an Si_3_N_4_ surface might be one of the causes of the decrease in the rate of proliferation of the cells. Both the behavior of PC12 cells on PLL coated Si_3_N_4_ surface in the presence of growth factors and the morphological changes these cells display are similar to those reported by Greene et al. [18] when they established this pheochromocytoma clonal cell line. The cells do not extend processes in serum-supplemented medium and can continue multiplying for an undetermined number of passages. After addition of NGF, we found that proliferation was partially inhibited and that differentiation began to occur in some cells. In the absence of serum and the presence of NGF, cell multiplication ceased after approximately 1 to 3 days of culture and the cells developed neurite-like processes. These observations are similar to the NGF response described by Greene et al. [18].

Letourneau [38] confirmed the importance of the interaction of chick embryonic sensory neurons with the substratum in a study using plastic tissue culture dishes that had been coated with various factors such as PLL. These factors strengthened the adhesion of the cells to the substratum and increased the probability of axon initiation and elongation and the degree of axonal branching. Moreover, coating plastic dishes with extracellular matrix produced by cultured corneal endothelial cells promoted rapid and tenacious attachment of PC12 cells to the coated surfaces triggering morphological differentiation even in the absence of NGF [30]. Our data indicate that the nature of the substrate was also important in addition to the PLL coating. Thus, cells seeded on the Si_3_N_4_ surface underwent enhanced morphological differentiation compared to those on a plastic dish. Moreover, the increase in the area of outgrowing extensions was significantly higher at all tested time points after 3 days of NGF stimulation for cells cultured on the Si_3_N_4_ surface. At 7 days after NGF stimulation, cells on the Si_3_N_4_ surface showed more than double the area of extensions as cells growing on the plastic dish. These observations confirm the importance of a strong adhesion of the cellular processes to the substratum to support increased differentiation [39]. Letourneau et al. [40] suggested that this effect might be due to the stabilization of the growth cone margin and that this stabilization promotes nerve fiber extensions through its influence on the organization of the microfilaments within the growth cone.

We used BrdU labeling to determine whether the ability of FBS to facilitate cell proliferation and of NGF to induce arrest of cell division and inhibit DNA synthesis differed between cells grown on an Si_3_N_4_ surface or a plastic culture dish. In medium supplemented with FBS, the rate of cell proliferation was lower on the Si_3_N_4_ surface compared to the plastic dish. This result is in agreement with our data obtained from counting the numbers of cells on both surfaces to assess their comparative rates of proliferation. The antagonistic effects of FBS and NGF on DNA synthesis are illustrated by the flattening of the curves for numbers of BrdU-labeled cells at 4 and 6 days after seeding cells in medium containing both FBS and NGF as compared to medium with FBS only. The binding of NGF to TrkA membrane-bound receptors induces tyrosine phosphorylation which leads to the activation of RAS/ERK signaling and to the development of a neuronal cell phenotype in differentiating PC12 cells [41], [42]. The cell proliferation curves in cultures containing both FBS and NGF were similar for both substrates although the numbers of cells on the Si_3_N_4_ surface were lower than on the plastic dish at every examined time point; this effect may have been produced by differences in NGF adsorption between the two surfaces. As cells were grown in the presence of FBS, they would have been continuously exposed to its stimulative effect for proliferation. Thus, this stimulative effect must have been counteracted to enable the cells leave the cell cycle and enter the quiescent state necessary for morphological differentiation.

On both substrates, cultures without FBS and horse serum but with NGF showed reduced cell proliferation in the absence of growth factors present in the sera**.** As a result, the proportion of cells undergoing DNA synthesis gradually fell due to the effect of NGF in the extracellular environment. However, even in the absence of sera and presence of NGF, the log phase of cell proliferation occurred one day after NGF stimulation in cells on both substrates; this response may be due to a limited cellular production of endogenous growth factors. Under the latter culture conditions, a higher rate of cells incorporating BrdU was observed during the first days of the experiment on the plastic dish; this difference between substrates might be explained by the inhibitory effect on proliferation of the interaction between the cells and the Si_3_N_4_ surface. We suggest that the fast reduction in cells entering S phase on the fifth day of culture on plastic dishes may have been due to the lack of the necessary serum components for growth and division. The anti-proliferative property of NGF may have added to this effect by inducing cells to remain in the G_0_ phase, or possibly more likely the G_1_ phase (just ready to overcome the G_1_ checkpoint with a positive proliferative stimulus). On the other hand, the numbers of BrdU-positive cells attached to the Si_3_N_4_ surface gradually decreased. We presume they became stationary in the G_0_ phase making them able to differentiate and to show a significantly longer survival time than cells cultured on the plastic dish. Possibly, the cell-substrate interaction of Si_3_N_4_ to maintain cells in the G_0_ phase compared to that of the plastic dish to hold them in the G_1_ phase (between withdrawal from the cell cycle for differentiation and entering the S phase for division) seems to be the determinant for the length of survival of the cells. As it was reported by Pucci et al. some proteins that function in proliferative pathways such as p53, RB and c-Myc may also act to sensitize cells to apoptosis, therefore affecting cell survival [43]. The Si_3_N_4_-cell interaction may offer another protective effect against cell death by preventing anoikis, a type of apoptosis induced by the lack of correct cell/ECM attachment [44]. It was previously reported that the topographical features of the PLL coated Si_3_N_4_ surface and its hydrophilicity make it a more suitable substrate for supporting cell attachment than a PLL coated plastic dish. The present analyses were performed in order to evaluate the properties of Si_3_N_4_ surface as a viable biomaterial for culturing neuron-like cells. Further research is clearly required to assess the proliferation/differentiation processes based on cell cycle analysis to identify the aspects of the cell division machinery affected by the contact with Si_3_N_4_ surface through selected coating molecules.

This research describes the functionality of Si_3_N_4_ for its use as an interfacial layer between biosensing devices and PC12 cells whose developmental properties and morphological resemblance to sympathetic neurons have made them widely used as a model cell line for the study of numerous problems in neurobiology and neurochemistry. The primary culture of neurons on Si_3_N_4_ will require a further conditioning of the surface in order to provide an extracellular environment close to the physiological one for the neurons to attach to the surface, differentiate and form synapses. However, a better understanding of PC12 cells behavior on this material may help us to adapt sensing surfaces for the culture of neurons as well as to improve the survival of these cells facilitating the performance of long-term sensing of molecular factors, an essential step for studying neuronal functions.

## Conclusion

The evaluation of Si_3_N_4_ as a substrate for cell-based biosensing devices will increase the prospect of development of new ion imaging sensors built upon ISFET technology that apply Si_3_N_4_ as a typical gate material. Here, we characterized the topographical features of the Si_3_N_4_ surface after conditioning for cell culture. Application of a poly-L-lysine coating increased the mean roughness and hydrophilicity of the material, thereby facilitating cell attachment. By comparing the behavior of neuron-like cells cultured on an Si_3_N_4_ surface and plastic dish, both coated with poly-L-lysine, we demonstrated that Si_3_N_4_, a biocompatible material conventionally used as the surface component of sensing LSI devices, can be also used as a cell culture supportive substrate. It is suitable for cell adhesion and enhances morphological differentiation in terms of growth of neurite extensions when cells are stimulated with a differentiation factor. Furthermore, we demonstrated that an Si_3_N_4_ surface possesses some functional advantages over conventional culture dishes made of polystyrene; for example, it promotes cell differentiation and lengthens cell survival time, important properties that make it ideal for the study of critical aspects of neuronal development and axonal guidance. Our BrdU incorporation assay showed that cells in contact with Si_3_N_4_ enter the quiescent state of the cell cycle preventing them undergoing DNA replication and consequently promoting an earlier and longer morphological differentiation compared to cells cultured on plastic dishes. This is the first report characterizing the cell-substrate interface of PC12 cells on a poly-L-lysine coated Si_3_N_4_ surface to be used for ISFET biosensing applications.

## Supporting Information

Figure S1 **Morphological and behavioral comparison between non-transfected and DsRed2-expressing PC12 cells.** PC12 cells (transfected and non-transfected) were seeded at a concentration of 7×10^4^ cells/ml on plastic cultures dishes. Differences in attachment (one day after seeding), proliferation (5 days after seeding in FBS-presence and NGF-absence condition) and differentiation (5 days after seeding in FBS-absence and NGF-presence condition) were assessed by observing the morphology and increase (non-quantified) in cell number and comparing these features between examined samples. PC12 cells transfected with DsRed2-encoding vector showed no obvious signs of reduced viability and displayed a similar morphology and increase in cell number corresponding to the conditions applied. Scale bar: 50 µm.
(TIF)

Figure S2 **Assessing morphological differentiation of PC12 cells by a standardized calculation of neurite growth, Area of extensions : Area of soma.** PC12 cells can differentiate into neuron-like cells following NGF stimulation. Outgrowths (lamellipodial or filopodial protrusions) elongate during differentiation. Comparison of the areas of these outgrowths to the area of the cell body provides an indication of the extent of differentiation. If the calculated ratio is less than 0.6, then it is assumed that no differentiation has occurred as small extensions are seen in unstimulated PC12 cells in long-term culture. If the calculated ratio is greater than 0.6, then it is concluded that the PC12 cell is morphologically differentiated. Scale bar: 10 µm.
(TIF)

Figure S3 **Effect of PLL coating degradation on PC12 cell attachment to an Si_3_N_4_ surface.** PC12 cells expressing DsRed2 protein were seeded (10×10^4^ cells/ml) onto 0.01% and 0.05% PLL coated Si_3_N_4_ surfaces previously incubated in extracellular solution for one and five days. Twenty four hours later, images were captured using a fluorescence microscope and attached cells were counted. The values shown are the mean ± S.E. of the number of cells counted in thirty images taken to six samples per group. A statistically significant difference between the number of cells attached to one day preincubated and five days preincubated PLL coated Si_3_N_4_ surfaces was found when using both PLL concentrations. (*, *p<*0.001 *vs.* one day preincubated PLL coated Si_3_N_4_ surface).
(TIF)

Figure S4 **PC12 cell attachment to surfaces widely used for cell culturing.** PC12 cells were seeded at the same concentration (7×10^4^ cells/ml) and under the same extracellular conditions (FBS-presence and NGF-absence) on different surfaces and 5 days later, images were captured. A representative image from each group was selected. The surfaces used as a substrate for cell culture were (A) PLL coated plastic dish, (B) non-coated plastic dish and (C) non-coated glass surface. Scale bar: 100 µm.
(TIF)
