# Supplementary figures and images for: Evaluation of Silicon Nitride as a Substrate for Culture of PC12 Cells: An Interfacial Model for Functional Studies in Neurons
Source: PLoS One. 2014 Feb 27;9(2):e90189. doi: 10.1371/journal.pone.0090189 (PMC3937378; doi:10.1371/journal.pone.0090189)

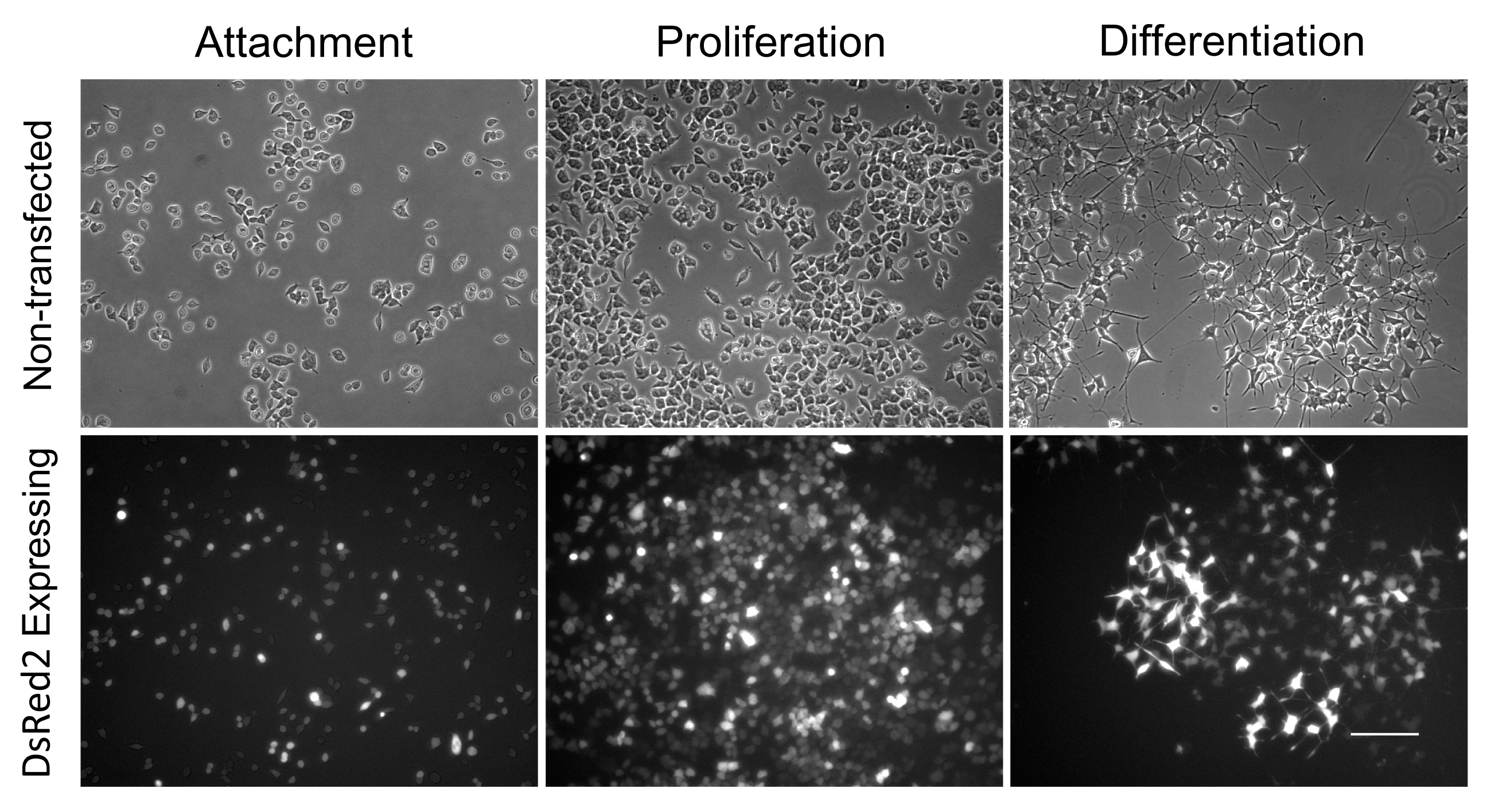

Supplement: Figure S1 — Morphological and behavioral comparison between non-transfected and DsRed2-expressing PC12 cells. PC12 cells (transfected and non-transfected) were seeded at a concentration of 7×10^4^ cells/ml on plastic cultures dishes. Differences in attachment (one day after seeding), proliferation (5 days after seeding in FBS-presence and NGF-absence condition) and differentiation (5 days after seeding in FBS-absence and NGF-presence condition) were assessed by observing the morphology and increase (non-quantified) in cell number and comparing these features between examined samples. PC12 cells transfected with DsRed2-encoding vector showed no obvious signs of reduced viability and displayed a similar morphology and increase in cell number corresponding to the conditions applied. Scale bar: 50 µm. (TIF) [file pone.0090189.s001.tif]

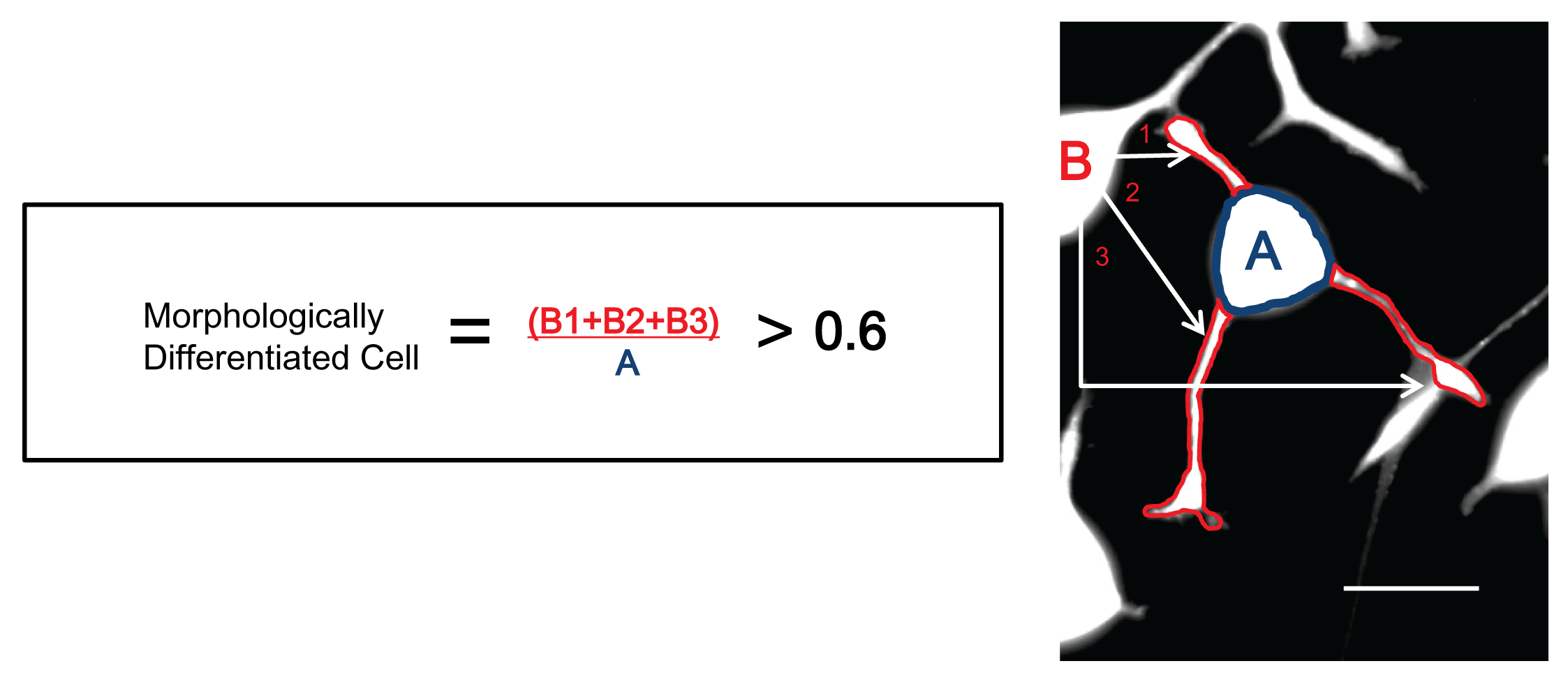

Supplement: Figure S2 — Assessing morphological differentiation of PC12 cells by a standardized calculation of neurite growth, Area of extensions : Area of soma. PC12 cells can differentiate into neuron-like cells following NGF stimulation. Outgrowths (lamellipodial or filopodial protrusions) elongate during differentiation. Comparison of the areas of these outgrowths to the area of the cell body provides an indication of the extent of differentiation. If the calculated ratio is less than 0.6, then it is assumed that no differentiation has occurred as small extensions are seen in unstimulated PC12 cells in long-term culture. If the calculated ratio is greater than 0.6, then it is concluded that the PC12 cell is morphologically differentiated. Scale bar: 10 µm. (TIF) [file pone.0090189.s002.tif]

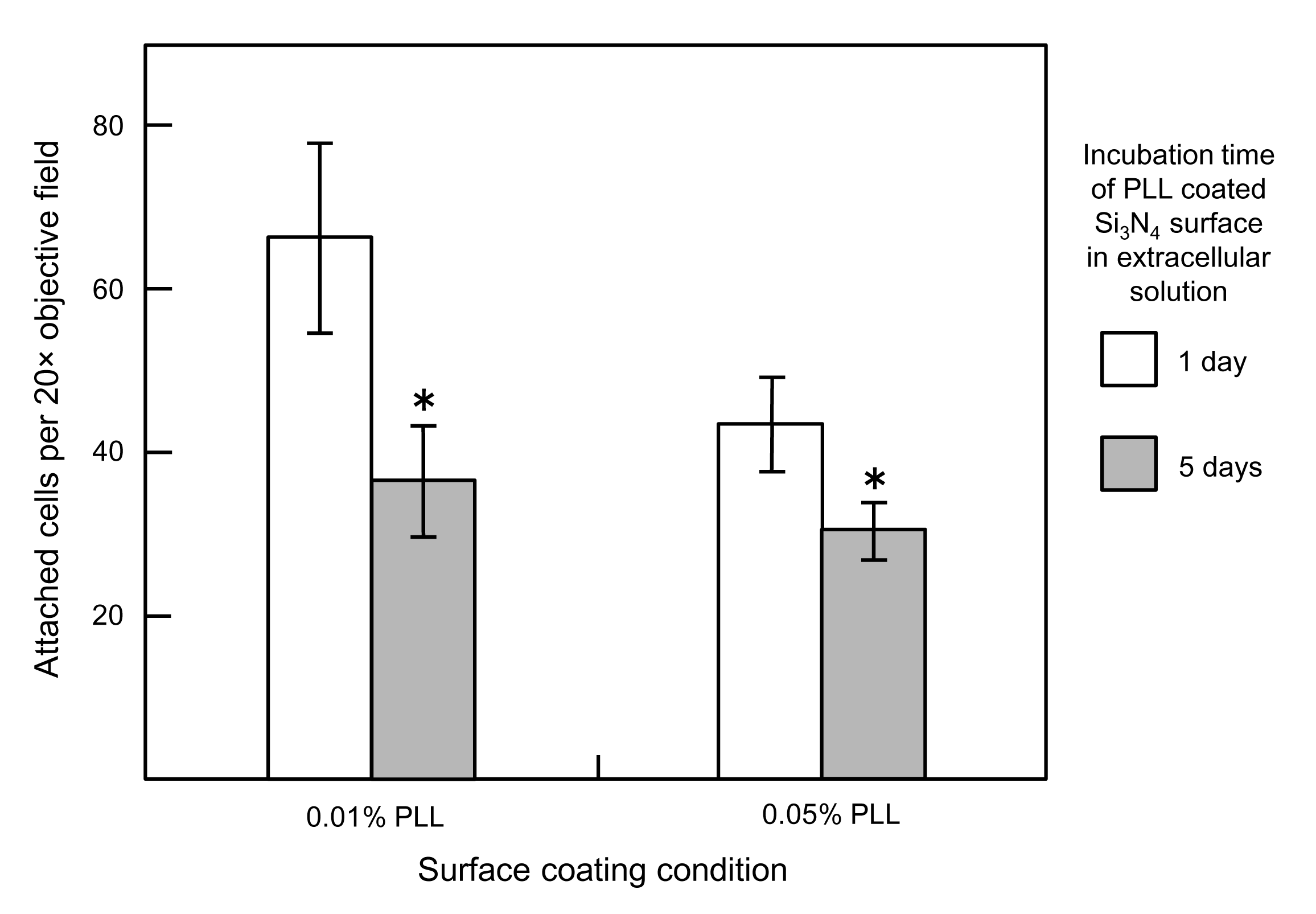

Supplement: Figure S3 — Effect of PLL coating degradation on PC12 cell attachment to an Si3N4 surface. PC12 cells expressing DsRed2 protein were seeded (10×104 cells/ml) onto 0.01% and 0.05% PLL coated Si3N4 surfaces previously incubated in extracellular solution for one and five days. Twenty four hours later, images were captured using a fluorescence microscope and attached cells were counted. The values shown are the mean ± S.E. of the number of cells counted in thirty images taken to six samples per group. A statistically significant difference between the number of cells attached to one day preincubated and five days preincubated PLL coated Si3N4 surfaces was found when using both PLL concentrations. (*, p<0.001 vs. one day preincubated PLL coated Si3N4 surface). (TIF) [file pone.0090189.s003.tif]

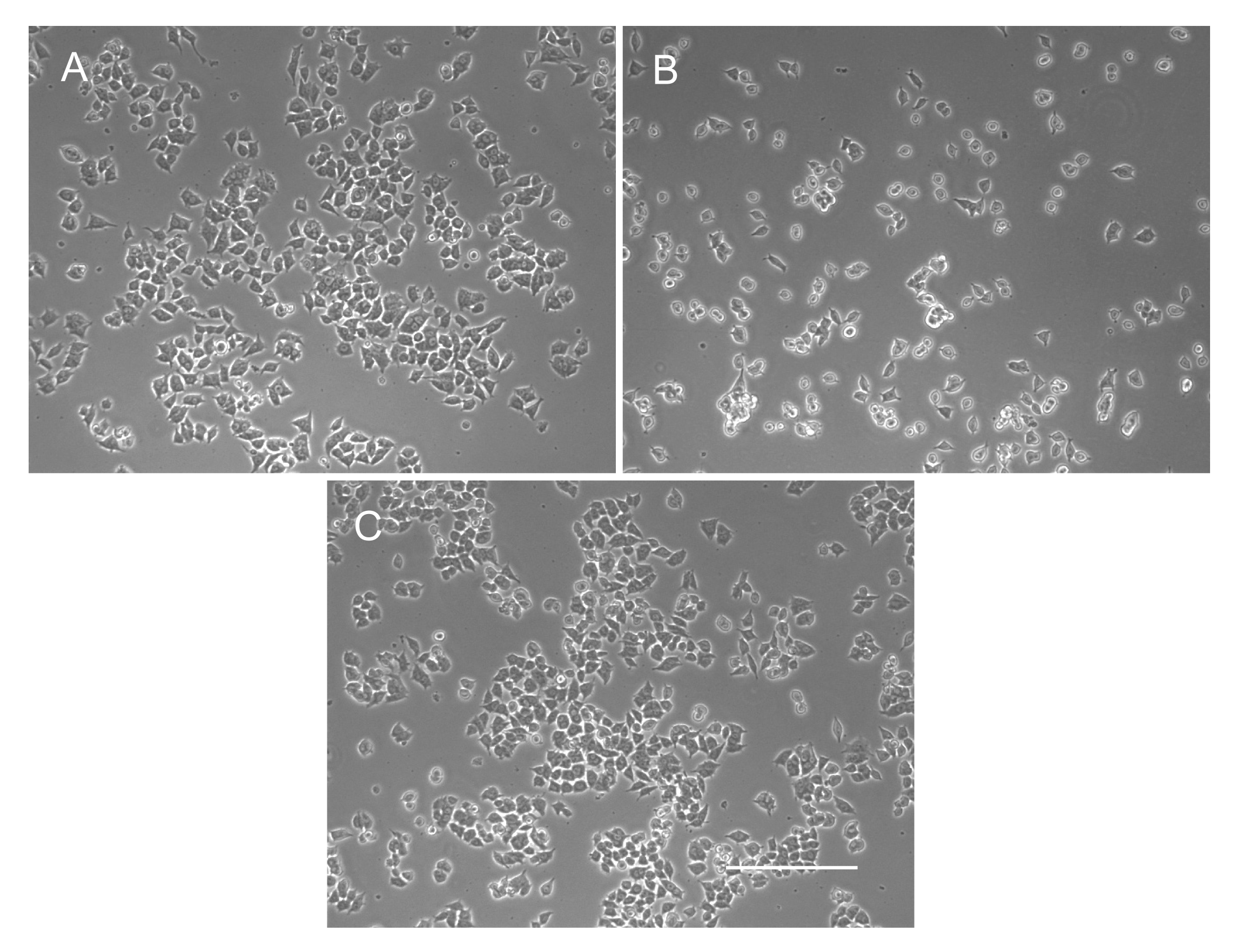

Supplement: Figure S4 — PC12 cell attachment to surfaces widely used for cell culturing. PC12 cells were seeded at the same concentration (7×10^4^ cells/ml) and under the same extracellular conditions (FBS-presence and NGF-absence) on different surfaces and 5 days later, images were captured. A representative image from each group was selected. The surfaces used as a substrate for cell culture were (A) PLL coated plastic dish, (B) non-coated plastic dish and (C) non-coated glass surface. Scale bar: 100 µm. (TIF) [file pone.0090189.s004.tif]
